# Fructo-oligosaccharides and glucose homeostasis: a systematic review and meta-analysis in animal models

**DOI:** 10.1186/s12986-018-0245-3

**Published:** 2018-01-25

**Authors:** Cindy Le Bourgot, Emmanuelle Apper, Sophie Blat, Frédérique Respondek

**Affiliations:** 1 R&D Department, Tereos, ZI et portuaire, 67390 Marckolsheim, France; 2 INRA, INSERM, Univ Rennes 1, Nutrition Metabolisms and Cancer (NuMeCan), Rennes, France

**Keywords:** Fructo-oligosaccharides, Prebiotic, Dietary fibres, Glycaemia, Insulinaemia

## Abstract

The aim of this systematic review was to assess the effect of fructo-oligosaccharide supplementation on glucose homeostasis. The search process was based on the selection of publications listed in the Pubmed-Medline database until April 2016 to identify studies evaluating the impact of short-chain fructo-oligosaccharides or oligofructose on glucose homeostasis. Twenty-nine trials were included in the systematic review and the meta-analysis was performed on twelve of these papers according to the inclusion criteria. Fasting blood concentrations of glucose and insulin were selected as pertinent criteria of glucose homeostasis for the meta-analysis. The consumption of fructo-oligosaccharides decreased fasting blood glycaemia levels, whatever the metabolic status (healthy, obese or diabetic) and diet (low-fat or high-fat) throughout the experiment. This reduction was linear with prebiotic dose (from 0 to 13% of the feed). Fasting insulinaemia also decreased linearly with fructo-oligosaccharide supplementation but the reduction was only significant in rodents fed a low-fat diet. Potential underlying mechanisms include gut bacterial fermentation of fructo-oligosaccharides to short-chain fatty acids (SCFA) and bacterial modulation of bile acids, both interacting with host metabolism.

This systemic review, followed by the meta-analysis, provides evidence that fructo-oligosaccharide supplementation has a significant effect on glucose homeostasis whatever the health status and diet consumed by animals.

## Background

Over the past decades, a food transition has taken place, characterized by the consumption of high energy density food. These dynamic changes in dietary macronutrient ingestion and lifestyle (increase in sedentarity) are leading causes of the growing prevalence of metabolic disorders such as obesity and type 2 diabetes. Emerging evidence suggests that bacterial dysbiosis within the gut may be one of the mechanisms of the pathogenesis of these metabolic diseases. Epidemiological and clinical studies have demonstrated that intake of dietary fibres, known for their promotion of more diverse/balanced intestinal microbiota, is inversely related to obesity [1] and type 2 diabetes [2]. Thus, the use of dietary fibre supplements to restore an optimal balance of intestinal microbiota may positively affect host metabolism, representing a potential beneficial strategy for individuals with metabolic disorders.

Dietary fibres are edible carbohydrate polymers, which are neither digested nor absorbed in the human small intestine [3], and have demonstrated beneficial physiological effect. There are different types of fibres, and according to their nature (naturally occurring in food, obtained from food raw material, or synthesized), physical properties, and fermentability in the gut, they do not have the same benefits for the consumer [4].

Most of the beneficial effects associated with high-fibre diets are linked to their low caloric value (2 kcal/g vs 4 kcal/g for other carbohydrates), their low postprandial glucose excursion (possibly due to their viscosity) [5] or their high content in mandatory micronutrients. Their fermentation by the gastrointestinal microbiota is also a cue of their beneficial effect on metabolism, especially due to their impact on glucose homeostasis [6]. Indeed, short-chain fatty acids (SCFA) produced by bacterial fermentation in the gut, enhance the release of GLP-1, an incretin secreted from intestinal L-cells [7]. Once released, GLP-1 binds to its specific receptor on pancreatic β-cells, stimulating insulin secretion, which participates in the regulation of glucose metabolism [8]. The continuous communication between the gut and the pancreas, that is mandatory to maintain glucose homeostasis, is named the entero-insular axis.

Several studies have indeed pointed out differences in the composition of the faecal microbiota of healthy people and patients with metabolic disorders, as recently reviewed [9].

β-fructans, composed of a terminal glucose molecule linked to fructose molecules by a β1–2 bound with varying degrees of polymerisation, are an example of such fermentable, non-viscous dietary fibres. Short-chain molecules of fructo-oligosaccharides (scFOS) and oligofructose (OF) are called fructo-oligosaccharides (FOS), while longer molecules are called inulin. Both are naturally present in various fruits and vegetables (including chicory), or can be produced from beet sugar. They are frequently used to replace sugars in the formulation of low-sugar foods in order to lower the postprandial glycaemic response [10, 11], to reduce energy content, or to enrich foods with dietary fibres. They are selectively fermented by a limited number of bacteria, especially *Bifidobacteria*, in the large intestine. Their health benefits for gut physiology in humans have been known for several years, but some recent studies point out that they may also have systemic benefits [12]. Yet, the capacity of FOS to lower fasting serum glucose in humans remains unclear [13], and further analysis of available animal studies would help to better understand the mechanism of action of FOS on glucose homeostasis and the relevance for human subpopulations that may benefit from these fibre supplementations.

This systematic review therefore focuses on the effects of scFOS and OF on glucose metabolism in animal models. A meta-analysis was then focused on the effect of dietary supplementation of these prebiotic fibres on fasting blood glycaemia and insulinaemia in rodents. We only included rodents in the meta-analysis due to the greater number of studies carried out on this animal model, and the larger number of animals used in each study compared to other animal species.

## Material and methods

### Systematic review

**Fig. 1:**
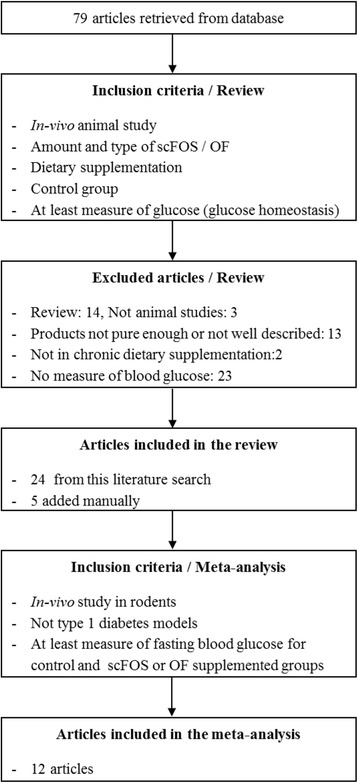
Flow diagram of the systematic literature search. Flow diagram showing the results of the systematic search for the selected studies in the systematic review and meta-analysis

For this review we defined short-chain fructo-oligosaccharides (scFOS) as molecules produced from beet sugar and having a degree of polymerization (DP) between 3 and 5, and oligofructose (OF) as molecules obtained from hydrolysis of inulin and having a DP between 2 and 8. All original research published in English and listed on PubMed-Medline until April 2016 using the following keywords: fructo(−)oligosaccharide(s), oligofructose and with one of the following words: blood glucose, glyc(a)emia, insulin, insulin(a)emia, diabetes, antidiabetic, metabolic syndrome were selected. The filter: “Other Animals” was applied. Seventy-nine articles were retrieved on this basis and they were selected for the review if they matched the described inclusion criteria (Fig. 1). Twenty-four articles were finally included from this search and 5 articles were manually added based on the same inclusion criteria.

### Meta-analysis

The aim of the meta-analysis was to focus on the effects of dietary supplementation with FOS (scFOS or OF) on fasting blood glycaemia and insulinaemia, indicative of metabolic health, in the studies in rodents retrieved from the systematic review. Selected papers for the meta-analysis therefore contained, at least, data on the physiological status of rodents (i.e. healthy, diabetic or obese), on diet characteristics and on blood parameters. Selected papers presenting more than one experiment were separated into experiments that were individually encoded.

Each comparison between control and prebiotic (scFOS or OF) was also individually encoded within each experiment containing more than 2 treatments. A total of 12 papers and 13 experiments were pooled in the database [14–25].

### Statistical analysis

The interpretation of this database was based on a statistical meta-analysis [26, 27]. The basic statistical model applied to the data was [26]:

$$ {Y}_{ijk}=\mu +{PRE}_i+{EXP}_j+{E}_{ijk}, $$

where Y_ijk_ = basal glycaemia (or insulinaemia); PRE_i_ = fixed effect of prebiotic; EXP_j_ = fixed effect of experiment j, and E_ijk_ = random residual error.

Prebiotic effect was first tested qualitatively (control vs FOS) and then as a covariable according to the dose of FOS supplementation expressed as % in diet. Normalized residuals greater than 3 were discarded from the analysis. The parameters of interest were the least square means (control vs FOS) or the adjusted equation (covariance analysis), the *P*-value for the FOS effect, and the outlier treatment that presented normalized residuals > 2. The fixed effect of experiment was always highly significant (*P* < 0.001) and is thus never provided. An effect was considered significant at *P* < 0.05.

## Results

### Glucose homeostasis

#### Systematic review

##### Database

We retrieved 29 studies evaluating the effects of a dietary supplementation with scFOS or OF on glucose homeostasis. Most of these studies was conducted in rodents (6 and 18 experiments in mice and rats respectively). The last 5 studies were performed in other animal species, namely veal calves (*n* = 1), horses (*n* = 1), dogs (*n* = 2) and cats (*n* = 1). The metabolic status of the animals was different between the experiments with either healthy, genetically obese or diet-induced obese (DIO), or genetically predisposed or streptozotocin-induced diabetic individuals. In addition, the type of diet used in the selected experiments varied regarding the energy provided by fat, and we characterized it as “low-fat” when fat provided less than 20% of the diet’s energy (standard diet), and as “high-fat” when fat provided more than 20% of the diet’s energy (high-fat diet), generally at the expense of carbohydrates. A synthetic description of the selected studies conducted in healthy rodents, or in obese or diabetic rodents, is presented in Tables 1 and 2 respectively. Papers on other species are described in Table 3.

**Table 1:** Effects of scFOS/OF supplementation on glucose homeostasis in healthy rodents

Animal model	Diet	FOS dose	Duration	Fasting condition		Postprandial condition			Other metabolic results	Microbiota analysis (yes/no)	Included in meta-analysis (yes/no)	References
				G	I	Method	G	I				
Standard diet: less than 20% of energy provided by fat												
Male Wistar rats	7% E fat, 73% E CHO	10% OF	4 w	ND	ND	Fed	↓	↓	↓ TG, PP (blood and liver) = tot. Chol (blood and liver)	NO	YES	[40]
Male Wistar rats	7% E fat, 73% E CHO	10% OF	4 w	=	↓	Fed (after 30d) OGTT (after 18d)	↓ (cardiac and portal blood) =	↓ (cardiac blood) ↓	↓ TG and ↑ GIP (blood) ↑ caecal GLP-1, = GIP	NO	NO	[41]
Male Sprague-Dawley rats	9.8%E fat, 77.5% E CHO	5% scFOS	3–5 w	=	↓ after 5 w	ND	ND	ND	↓ adiponectin, leptin ↓ HOMA-IR after 5 w = TG, MCP-1, PAI-1	NO	YES	[25]
Male Wistar rats	9.8% E fat, 77.5% E CHO	5% OF 10% OF	10 w	= =	↓ ↓	ND	ND	ND	↓ HOMA-IR ↑ RBC glucose transmembrane transport	NO	YES	[21]
Male Wistar/ST rats	9.8%E fat, 77.5% E CHO	5% scFOS	48 d	=	=	OGTT	=	=	= HOMA-IR	NO	YES	[23]
Male Wistar rats	11.8% E fat, 69.4% E CHO (starch or fructose)	10% OF	4 w	ND	ND	Fed	=	=	↓ TG ↓ leptin with fructose diet only	YES	YES	[61]
Female C57BL/6 J mice ± n-3 PUFA	14.3% E fat, 58.9% E CHO	0.2 g OF/ d	24 d	↓ (in n-3 depleted mice only)	=	OGTT	=	=	= HOMA-IR ↑ colon proglucagon mRNA content = hepatic TG and Chol	YES	YES	[22]
Male Sprague-Dawley rats	16.4% E fat, 65.1% E CHO	2.5% or 5% scFOS	7 w	= (tail and portal blood)	= (tail blood) ↓(portal blood) with 5% scFOS	IPGTT	↓ (2.5% at 30 min)	ND	= HOMA-IR ↑ portal fasting GLP-1 (5%) ↑proglucagon mRNA in caecum and colon (5%) = GLP-1 in ileum, caecum and colon	NO	NO	[58]
High-fat diet: 20 to 60% of energy provided by fat												
Male Wistar rats	23.9% E fat, 56.8% E CHO	5% OF	3 w	ND	ND	Fed^a^	=	=		NO	NO	[83]
Male Sprague-Dawley rats	27.5% E fat, 53.5% E CHO	10% scFOS	3 w	ND	ND	Fed	↓	=	= plasma acetate, propionate, butyrate ↓ FFA and TG = tot. Chol, PP	NO	YES	[38]
Male C57BL/6 J gnotobiotic mice	34.9% E fat, 26.3% E CHO	10% scFOS	6 w	ND	ND	Fed (5 h after meal) OGTT	= = AUCg	= = I (20 and 60 min)	↓ leptin	YES	NO	[46]
Male C57BL/6 J mice	Exp 2 HF2: 58% E fat, 26% E CHO	10% OF	4–8 w	=	=	ND	ND	ND	= colon proglucagon mRNA	NO	YES	[16]
High-fat diet: more than 60% of energy provided by fat												
Male C57BL/6 J mice	72% E fat, < 1% E CHO	10% OF	4 w	↓	↑	Fed IPGTT	↓ ↓ AUCg	↑ ND	↑ pancreatic insulin ↑ glucose infusion rate and ↓ hepatic glucose production (clamp euglycaemic hyperinsulinaemic) ↑ colon proglucagon mRNA ↑ GLP-1 (plasma and colon)	NO	YES	[15]
Male C57BL/6 J mice	Exp 1 HF1: 72% E fat, < 1% E CHO	10% OF	4–8 w	↓	↑	ND	ND	ND	↑ colon proglucagon mRNA	NO	YES	[16]

*CHO* carbohydrates, *Chol* cholesterol, *d* days, *E* energy, *FFA* free fatty acids, *FOS* fructo-oligosaccharides, *G* glucose, *I* insulin, *MCP-1* monocyte chemoattractant protein-1, *ND* no data, *OF* oligofructose, *PAI-1* plasminogen activator inhibitor-1, *PP* phospholipids, *PUFA* polyunsaturated fatty acids, *RBC* red blood cells, *scFOS* short-chain fructooligosaccharides, *TG* triglycerides, *w* weeks

^a^Fed condition not clearly indicated in the study

**Table 2:** Effects of scFOS/OF supplementation on glucose homeostasis in obese or diabetic rodents

Animal model	Diet	FOS dose	Duration	Fasting condition		Postprandial condition			Other metabolic results	Microbiota analysis (yes/no)	Included in meta-analysis (yes/no)	References
				G	I	Method	G	I				
Standard diet: less than 20% of energy provided by fat												
Male Obese *fa/fa* Zucker rats	7% E fat, 73% E CHO	10% OF	10 w	=	=	- Fed (tail vein) - OGTT - Fed (cava and portal veins)	- = - = - ↓	- ND - = - =	= PP, TG and Chol (cava and portal veins) ↓ hepatic TG and PP	NO	YES	[39]
Female Obese Zucker rats	9.8% E fat, 77.5% E CHO	5% scFOS	100 d	=	↓	ND	ND	ND	= TG and Chol = HbA1C	NO	YES	[20]
Male Sprague-Dawley rats, DIO	9.8% E fat, 77.5% E CHO	3% OF	6 w	=	=	OGTT	=	=	↑ PYY OGTT and AUC PYY = GLP-1 = liver TG	YES	YES	[17]
Male Sprague-Dawley rats, DIO	9.8% E fat, 77.5% E CHO	10% OF	8 w	↓	=	OGTT	↓ 90 min	↓	↓ leptin OGTT (AUC leptin) ↑ fasting PYY and AUC PYY ↑ fasting portal GLP-1	YES	YES	[14]
Diabetes-prone BB rats	16.4% E fat, 65.1% E CHO	5% OF	160 d	=^a^	ND	ND	ND	ND	ND	NO	NO	[84]
Male Wistar rats – Streptozotocin	7% E fat, 73% E CHO	10% OF	6 w	ND	ND	- OGTT - Fed	- ↓ - ↓	- ↑ - ↑	↑ pancreatic insulin and % beta cells (= beta cell mass) ↑ portal and colon GLP-1 ↑ colon proglucagon and PC1 mRNA (= in ileum)	NO	YES	[37]
Male Wistar rats – PX - 407	9.8% E fat, 77.5% E CHO	10% or 15% scFOS	6 w	ND	↑	ND	ND	ND	↑ caecal GLP-1	YES	NO	[56]
High-fat diet: 20 to 60% of energy provided by fat												
Male Sprague Dawley rats, DIO	g/100 g: casein (20.0), sucrose (49.9), soybean oil (10.0), lard (10.0)	10% OF	7 w	ND	ND	OGTT	↓	=	↓ leptin, ghrelin, GIP OGTT ↑ GLP-1 OGTT ↓ plasma DDP4	YES	YES	[24]
Male Wistar rats – Streptozotocin	20.4% E fat, 59.9% E CHO	10% scFOS	2–6 w	↓ (after 2, 4 and 6 w)	ND	ND	ND	ND	↓ urinary glucose excretion (after 4, 5 and 6 w) ↓ plasma cholesterol, creatinine and urea	YES	YES	[19]
High-fat diet: more than 60% of energy provided by fat												
*Ob/ob* C57BL/6 mice	60% E fat, 20% E CHO	10% OF	5 w	↓	ND	OGTT	↓	ND	↓ plasma TG, LPS ↑ plasma GLP-1 and colon proglucagon mRNA ↑ colon L-cells number	YES	YES	[18]

*AUC* area under the curve, *CHO* carbohydrates, *Chol* cholesterol, *d* days, *DIO* diet-induced obesity, *DPP4* dipeptidyl peptidase-4, *E* energy, *FOS* fructo-oligosaccharides, *G* glucose, *I* insulin, *LPS* lipopolysaccharide, *ND* no data, *OF* oligofructose, *PP* phospholipids, *scFOS* short-chain fructooligosaccharides, *TG* triglycerides, *w* weeks

^a^Fed condition not clearly indicated in the study

**Table 3:** Effects of scFOS/OF supplementation on glucose homeostasis in animals other than rodents

Animal model	Diet	FOS dose	Duration	Fasting condition		Postprandial condition			Other metabolic results	Microbiota analysis (yes/no)	Included in meta-analysis (yes/no)	References
				G	I	Method	G	I				
Healthy male veal calves	Whole milk + milk replacer	10 g scFOS/d	3 w	=	=	Lactose feeding	↓ (after 2 h to 5 h)	↑ (after 2 h)	↓ lactate after lactose feeding (after 3 h and 4 h) = TG (blood)	NO	NO	[29]
Obese male Arabian horses	Concentrate feeds and hay	45 g scFOS/d	6 w	=	↓	FSIGTT	= Sg	↓ AIRi ↑ SI	= TG and leptin (fasting blood)	NO	NO	[28]
Obese male Beagle dogs	32% E fat, 44% E CHO	1% scFOS	6 w	=	=	Euglycemic hyperinsulinaemic clamp	↑ glucose infusion	↑ insulin sensitivity	↓ HOMA-IR = TG and Chol (blood)	NO	NO	[30]
Obese male Beagle dogs on weight-loss program	9.5% E fat, 56.5% E CHO	3% scFOS Vs 1% scFOS	6 w	=^a^	=^a^	ND	ND	ND	↓ haptoglobin	NO	NO	[31]
Normal or obese short-hair neutered cats	38% E fat, 8% E CHO	2.5% mix OF/inulin	4 w	=	=	IVGTT	=	=	= Chol, NEFA, leptin	NO	NO	[32]

*CHO* carbohydrates, *Chol* cholesterol, *d* days, *E* energy, *FOS* fructo-oligosaccharides, *G* glucose, *I* insulin, *ND* no data, *OF* oligofructose, *scFOS* short-chain fructooligosaccharides, *TG* triglycerides, *w* weeks

^a^Fed condition not clearly indicated in the study

##### Glucose homeostasis parameters

In studies conducted in healthy rodents fed with standard diets, FOS supplementation generally did not affect blood fasting glucose concentration. Although fasting glycaemia was not changed, fasting blood insulin was lowered in 4 out of the 6 studies where it was measured. FOS supplementation reduced postprandial blood glucose level in half of the studies, in association with decreased insulin concentration when measured. When rodents were fed a high-fat diet (more than 60% of energy provided by fat), supplementation with FOS significantly increased fasting insulinaemia and decreased in parallel fasting glycaemia, and the same results were obtained in the fed state (Table 1). In rodents with obesity or type 2 diabetes, the same tendency towards reduction of glycaemia was observed with FOS supplementation, particularly in postprandial condition. Effects on insulinaemia were more controversial, with either a reduction in obese rats or an increase in diabetic rat models (Table 2).

In addition, five studies on animal models other than rodents (i.e. veal calves, Arabian horses, Beagle dogs, neutered short-hair cats) were retrieved from the literature search (Table 3). Contrary to studies involving rodents, most of these studies were conducted in a cross-over design and no study was performed with a high-fat diet for the considered species, even though the percentage of energy provided by fat in the diet of dogs and cats was around 30%, considered as high for rodents but normal for pets. In these studies, scFOS supplementation had no effect on fasting blood glucose and insulin, except for the study with obese horses in which it reduced fasting blood insulin [28]. In these species, the effects of scFOS on glucose homeostasis were more visible in postprandial state or in a dynamic model of glucose tolerance test. In veal calves, scFOS supplementation decreased the postprandial glucose response to a lactose-rich meal and increased postprandial insulin secretion [29]. Two recent studies on obese and insulin resistant horses [28] and dogs [30], showed that a 6-week dietary supplementation with scFOS could improve insulin sensitivity with no change in body weight. In horses, the frequently sampled intravenous glucose tolerance test (FSIGTT) highlighted that this improvement in insulin sensitivity was accompanied by a reduction in acute insulin response to glucose, with no change in glucose effectiveness. When obese dogs were submitted to a weight loss program with an energy-restricted diet, no effect of scFOS supplementation was observed on fasting blood glucose or insulin [31]. In normal weight and obese cats, the diet supplementation with a mixture of OF and inulin did not affect glucose homeostasis in fasting condition and after an IV glucose bolus [32].

### Meta-analysis

The meta-analysis was performed on 12 papers and 13 experiments. It represented 32 treatments for the fasting glycaemia parameter, and 14 treatments for fasting insulinaemia (Table 4).

**Table 4:** Meta-analysis of the FOS supplementation effect on fasting blood glucose and insulin concentrations in rodents

Parameter	Metabolic health or diet	Treatment	N	Mean ± SEM	Minimum value	Maximum value	*P*-value Treatment
Fasting blood glucose, *mmol/l*	Healthy + unhealthy	Control	15	10.6 ± 1.6	4.8	25.2	0.0124
		FOS	17	8.7 ± 1.3	4.6	26.3	
	Healthy	Control	7	6.8 ± 0.4	4.8	8.3	0.002
		FOS	9	6.1 ± 0.4	4.6	8.1	
	Unhealthy (obese or diabetic)	Control	8	13.9 ± 2.6	5.5	25.2	0.0398
		FOS	8	11.5 ± 2.5	4.7	26.3	
	Low-fat (< 20%E)	Control	7	8.9 ± 2.7	4.8	25.2	0.0083
		FOS	9	8.0 ± 2.3	4.6	26.3	
	High-fat (> 20%E)	Control	8	12.0 ± 1.9	5.7	18.3	0.0225
		FOS	8	9.4 ± 1.3	4.7	13.4	
Fasting blood insulin, *pmol/l*	Healthy + unhealthy	Control	6	155.3 ± 29.4	73.7	253	0.0936
		FOS	8	116.1 ± 23.8	52.6	225	
	Healthy	Control	5	135.8 ± 26.9	73.7	214.8	0.5922
		FOS	7	122.6 ± 26.5	52.6	225	
	Unhealthy (obese or diabetic)	Control	1	253.0	–	–	–
		FOS	1	70.6	–	–	
	Low-fat (< 20%E)	Control	3	160.1 ± 50.9	77.4	253	0.0136
		FOS	5	74.4 ± 10.1	52.6	110	
	High-fat (> 20%E)^a^	Control	3	150.5 ± 41.2	73.7	214.8	0.2159
		FOS	3	185.7 ± 32.8	120.5	225	

*E* energy, *FOS* fructo-oligosaccharides

^a^Only on healthy animals (not enough data on unhealthy animals)

#### Qualitative analysis: control vs FOS

FOS supplementation significantly decreased fasting blood glycaemia, whatever the metabolic status of the rodents and the diet administered throughout the experiment (*P* = 0.012; Table 4), with a global reduction of 18% of fasting glycaemia in the FOS group compared to the control group. The greater reduction in fasting blood glucose with FOS intake was observed in rodents fed a high-fat diet (− 22%). In parallel, a decrease in fasting insulinaemia was only observed when comparing control and FOS groups fed a low fat diet (*P* = 0.014).

#### Quantitative analysis: effect of FOS dose

A consistent effect with the qualitative model was obtained with the linear model (Table 5). Fasting glycaemia decreased linearly with FOS dose (0 to 13%), whatever the metabolic health and the diet administered throughout the experiment (− 0.17 mmol/L per 1% FOS supplementation; *P* = 0.002; Table 5). A significant dose effect was also observed for basal insulinaemia (− 6.46 pmol/L per 1% FOS supplementation; *P* = 0.04), particularly in healthy rodents (P = 0.002) and in rodents fed a low-fat diet (*P* = 0.016), with no significant difference in animals fed a high-fat diet (*P* = 0.22), probably due to the small volume of available data (Table 5).

**Table 5:** Meta-analysis of the effect of FOS dose supplementation on rodents fasting blood glycaemia and insulinaemia

Parameter	Metabolic health or diet	N	Intercept	Slope	*P-*value Dose	SD
Fasting blood glucose, *mmol/l*	Healthy + unhealthy	32	9.74	−0.17	0.0022	0.32
	Healthy	16	6.84	−0.085	0.0007	0.012
	Unhealthy (obese or diabetic)	16	12.98	−0.243	0.012	0.566
	Low-fat (< 20%E)	16	8.93	− 0.035	0.0035	0.012
	High-fat (> 20%E)	16	10.48	−0.261	0.0054	0.526
Fasting blood insulin, *pmol/l*	Healthy + unhealthy	14	154.4	−6.46	0.0386	7.02
	Healthy	12	134.74	−0.459	0.0022	7.67
	Unhealthy (obese or diabetic)	–	–	–	–	–
	Low-fat (< 20%E)	8	158.4	−16.45	0.0159	9.79
	High-fat (> 20%E)	6	150.5	3.52	0.216	13.90

*E* energy, *FOS* fructo-oligosaccharides

### Other metabolic effects

In addition to glucose and insulin concentration data, most authors also studied incretin effect and lipid profile after FOS supplementation in rodents (Tables 1 and 2). In most of the studies, FOS supplementation caused an increase in intestinal GLP-1 and proglucagon mRNA content. FOS supplementation also increased fasting and postprandial GLP-1 concentrations, as well as PYY concentration when analysed. FOS supplementation decreased triglyceride concentration in half of the studies. Cholesterol reduction with prebiotic intake was less consistent. Two studies reported a reduction in plasma inflammatory markers with FOS supplementation: LPS in obese mice fed a high-fat diet, and haptoglobin in obese dogs subjected to a weight-loss program (Tables 2 and 3).

### Microbiota modifications

Some studies also analysed microbiota composition and/or fermentative activity (Table 6). In response to FOS consumption, the weight of caecum (tissue and/or content) was increased, reflecting a higher fermentative activity of the microbiota. When measured, short-chain fatty acid (SCFA) content was increased in the FOS-supplemented group, and particularly propionate and butyrate. Microbiota composition was also modified by FOS supplementation and notably by an increase in *Bifidobacterium*, *Lactobacillus* and *Clostridium coccoides*, and by a reduction in *Clostridium leptum*. Effects on *Bacteroides/Prevotella* ratio were more controversial (Table 6).

**Table 6:** Effects of scFOS/OF supplementation on microbiota composition and/or fermentative activity

Animal model	Diet	FOS dose	Duration	Microbiota fermentative activity	Microbiota composition	References
Male Wistar rats	11.8% E fat, 69.4% E CHO (starch or fructose)	10% OF	4 w	↑ caecum weight ↑ caecal SCFA (pool) content: ↓ acetate, ↑ propionate and butyrate	ND	[61]
C57BL/6J female mice, depleted or not in n-3 PUFA	14.3% E fat, 58.9% E CHO	0.2g OF/ d	24 d	↑ caecal tissue and content weight	Caecum (STD diet): ↑*Bifidobacterium* spp., *Bacteroides-Prevotella* ↓ *Lactobacillus* spp. Caecum (n-3 depleted diet): ↑*Bifidobacterium* spp., *Lactobacillus* spp. = *Bacteroides-Prevotella*	[22]
Male C57BL/6J gnotobiotic mice	34.9% E fat, 26.3% E CHO	10% scFOS	6 w	↑ empty and full caecum weight	Faeces: ↑ Bifidobacteria, *C. coccoides* ↓ *C. leptum, r*atio *Bacteroides-Prevotella*: *C. coccoides*	[46]
Male Sprague-Dawley rats, DIO	9.8% E fat, 77.5% E CHO	3% OF	6 w	ND	Caecum: ↑ Total bacteria ↑ *Lactobacillus*, *Bifidobacterium* and *Bacteroides/Prevotella*, ↓ *C. leptum*, *C. cluster XI*, ↓ % Firmicutes, ratio Firmicutes: Bacteroidetes	[17]
Male Sprague-Dawley rats, DIO	9.8% E fat, 77.5% E CHO	10% OF	8 w	↑ caecum weight	Caecum: ↑ *Bacteroides* spp., *Lactobacillus* spp., *Bifidobacterium* spp., *B. animalis* ↓ *C. coccoides*, *C. leptum*, *Clostridium Cluster XI and I*, *Enterobacteriaceae* ↓ Ratio Firmicutes: Bacteroidetes	[14]
Male Wistar rats – PX - 407	9.8% E fat, 77.5% E CHO	10% or 15% scFOS	6 w	ND	Caecum: ↑ Bifidobacteria and Lactobacilli	[56]
Male Sprague Dawley rats, DIO	g/100g: casein (20.0), sucrose (49.9), soybean oil (10.0), lard (10.0)	10% OF	7 w	↑ empty caecum weight	Faeces: ↑ Total bacteria ↑ *Bifidobacterium* spp. ↓ *C. leptum* *= Lactobacillus* spp.*, C. coccoides, Bacteroides/Prevotella*	[24]
Male Wistar rats, Streptozotocin	20.4% E fat, 59.9% E CHO	10% scFOS	2 – 6 w	↑ caecum weight	Caecum: ↑ Bifidobacteria, Lactobacilli	[19]
*Ob/ob* C57BL/6 mice	60% E fat, 20% E CHO	10% OF	5 w	↑ caecum and colon weight	Caecum (qPCR): = Total bacteria *↑ Bifidobacterium* spp., *E. rectale/* *C. coccoides* group ↓ Firmicutes and *Roseburia* spp. = *Bacteroidetes*, *Lactobacillus* spp., *Bacteroides-Prevotella* group	[18]

*CHO* carbohydrates, *d* days, *DIO* diet-induced obesity, *E* energy, *FOS* fructo-oligosaccharides, *G* glucose, *I* insulin, *ND* no data, *OF* oligofructose, *scFOS* short-chain fructooligosaccharides, *w* weeks

## Discussion

The aim of the current paper was to undertake a systematic review and a meta-analysis of animal studies to evaluate the effect of FOS supplementation on glucose homeostasis. Overall, the results from rodent studies showed that regular consumption of FOS significantly reduces fasting glycaemia compared to non-supplemented animals, whatever the metabolic status of the animals and their type of diet. The range of FOS supplementation was 5 to 13% of the total diet and its duration was between 2 to 14 weeks. Interestingly the reduction in fasting glycaemia was more pronounced when rodents were fed a high-fat diet (− 22%) compared to a low-fat diet (− 10%) and when rodents were obese or diabetic (− 17%) compared to healthy (− 10%). These results suggest that the effects of FOS supplementation are more pronounced in the event of glucose homeostasis failure in rodents.

Effects of FOS supplementation on fasting insulinaemia were inconsistent. In the meta-analysis, a trend towards lower fasting insulinaemia with FOS supplementation was obtained when unhealthy and healthy rodents were gathered (Table 4); however, this parameter was strongly influenced by the single study made on obese rats in which fasting insulinaemia was reduced by 73% [20]. The effect of FOS supplementation on fasting insulinaemia seemed to be dependent on the type of diet given to the rodents, with a significant effect of FOS supplementation on fasting insulin decrease in rodents fed a standard diet only (less than 20% of energy provided by fat). However, there were only few data available for a high-energy diet, which may have contributed to the lack of significance of FOS supplementation effect with this type of diet in rodents.

In other animal models than rodents, no effect of FOS on fasting glycaemia has been observed as seen in cats [32], dogs [30, 31], horses [28], and veal calves [33]. On the other hand, fasting insulinaemia decreased in obese horses with FOS supplementation [28], and did not change in other species [32, 33]. Similarly, a recent meta-analysis performed on 26 trials involving 831 humans [34] consuming all types of prebiotics did not show significant difference in fasting glycaemia and reported inconsistent results on fasting insulinaemia. This inconsistency of the results could be due to a lack of standardization of the insulin assay procedure, making it hard to compare absolute plasma insulin concentration values from one laboratory to another [35]. Thus, fasting insulinaemia seems to be a poor parameter for evaluating the effect of FOS supplementation on glucose homeostasis.

Yet, the lowering effect of a FOS supplementation on fasting glycaemia is well demonstrated in rodents, while things are less clear with the other animal models or in humans. Many more studies and data are available in rodent models compared to other animal models and data in humans, as well as the higher dose of supplementation used in rodents, may partly explain why FOS supplementation has demonstrated significant effects in rodents only, the number of studies in other species being too low to underpin any significant effect. In addition, a recent review suggested that postprandial glycaemia was a better predictor of overall glycaemic control than fasting glycaemia [36].

In postprandial conditions such as fed state, or after a glucose homeostasis challenge with a glucose bolus, FOS supplementation reduced glycaemic response in most of the studies selected in our review, though with various effects on insulinaemia. In our bibliography research on rodents, decreased postprandial glucose was concomitantly observed with either an increase [15, 37], no effect [18, 38, 39], or a decrease [14, 40, 41] in postprandial insulinaemia. It is worth mentioning that increased postprandial insulinaemia with FOS supplementation was observed in two studies conducted in diabetic rodents (induced by HF diet or treatment) where glucose-stimulated insulin secretion was significantly reduced [15, 37]. Under these specific conditions, FOS supplementation made it possible to normalize postprandial insulin concentration, bringing it in line with that of non-diabetic rodents. Two studies showed an improvement in insulin sensitivity in obese dogs and horses [28, 30]. The meta-analysis performed in humans indicated a statistically significant effect of FOS supplementation on postprandial glucose and insulin, supporting the fact that prebiotic consumption results in a reduction of both postprandial glucose and insulin concentrations [34]. Altogether, β-fructan improves glucose homeostasis by different mechanisms, depending on metabolic status.

Even though the effects on glycaemia were the same under fasting and postprandial conditions in rodents, the results obtained in dynamic model reflect the adaptability of the organism. Indeed, the dysglycaemia worsening process in type 2 diabetes, for example, is marked first by an early loss of postprandial glycaemic control, preceding a deterioration of fasting glycaemia leading to chronic sustained fasting hyperglycaemia. So, the results obtained in the dynamic model are probably earlier markers of the effect of FOS on glucose metabolism than fasting values. Moreover, it has been recommended to take into account the respective contribution of both fasting and postprandial conditions for assessing glucose homeostasis [42]. However, as dynamic models may be invasive, finding “easy-to-sample” markers is useful for the evaluation of the prebiotic efficiency in practical life.

The gut microbiota has emerged as an integral factor that impacts host metabolism with some evidence for its direct involvement in insulin sensitivity. Also, considering this interplay between gut microbiota and host insulin sensitivity [43], the effects of scFOS/OF on glucose homeostasis presented here probably occurred through microbiota modulations. FOS are prebiotic fibres selectively fermented by the microbiota inhabiting the large intestine [12]. While scFOS and OF may not influence the composition of the microbiota in exactly the same way [44], their fermentation would generally stimulate the growth of some *Bifidobacteria* and *Lactobacilli* groups (Table 6), and also directly or indirectly stimulate the growth of lactate-utilizing bacteria [44, 45] and *Clostridium coccoides* [46]. The fermentation of FOS by the intestinal microbiota is generally complete, leading to an increase in SCFA production [47–53], and more particularly a long-term increase in faecal butyrate, sometimes following a transient increase in lactate production [48]. The mechanisms linking microbiota and glucose homeostasis have been partly elucidated, notably thanks to studies in rodents, and this includes modulation of host signalling through bacterial fermentation products such as SCFA. Indeed, SCFA can bind to the G protein-coupled receptors GPR41 and GPR43, which are widely expressed on intestinal epithelial, enteroendocrine and immune cells, but also in other metabolically important tissues such as adipose tissues, liver and pancreas [54]. For instance, acetate and propionate, and propionate and butyrate, through GPR43 and GPR41 binding respectively on intestinal enteroendocrine cells, regulate the secretion of intestinal hormones involved in glucose metabolism regulation. Tolhurst et al. (2012) demonstrated that SCFA, and particularly acetate and propionate, enhanced the release of GLP-1 in an in vitro model of colonic culture. They also showed that mice lacking gpr41 or gpr43 exhibited reduced SCFA-triggered GLP-1 secretion in vivo and developed impaired glucose tolerance, highlighting the important role of SCFA on glucose metabolism through GLP-1 secretion [7]. Interestingly scFOS supplementation (5% for 4 weeks) increased the density of GPR43-positive enteroendocrine cells in rat proximal colon by over two-fold in comparison to control non-supplemented rats, in parallel to an increased density of GLP-1 containing L-cells [55]. Therefore, GPR43 activation by SCFA might be an important trigger for the production and release of GLP-1. In our literature review, scFOS or OF supplementation reduced post-prandial glucose with, in most studies, a concomitant increase in GLP-1 concentration together with a higher number of enteroendocrine L-cells, both in healthy and obese or diabetic rodents, whatever the pattern of post-prandial insulinaemia [14, 15, 18, 24, 41, 56–58]. These data are in agreement with a microbiota – host metabolism interplay involving GLP-1 release. In addition, SCFA are involved in the regulation of lipid metabolism and adipose tissue [59]. For example, a butyrate supplementation decreased the blood concentration of cholesterol and triglycerides in mice fed HF diet [60], in accordance with the study of Busserolles et al. (2003) included in our systematic review, where an increase in butyrate content in the caecum paralleled a reduction of plasma triglycerides in rats supplemented with OF [61]. Furthermore, a meta-analysis conducted in rodents confirmed the possible benefits of scFOS to reduce visceral fat mass deposition that is a risk factor for metabolic disorders as type-2 diabetes [62]. This suggests inter-relationships between lipid and glucose metabolisms involving microbiota changes. However, the inconsistent results on blood cholesterol and triglycerides in our systematic review require more investigations.

Other signaling pathways mediating crosstalk between gut bacteria and host glucose homeostasis have been identified, through the production of bile acids for instance [63]. Several studies have shown that the gut microbiota impacted bile acid metabolism and signalling by biotransforming bile acids through deconjugation, dehydroxylation, and reconjugation [64]. This may regulate the bile acid pool and composition. Primary bile acids are produced by the liver and recirculated to the liver from the gut. The primary deconjugated/dehydroxylated bile acids are further metabolized by gut bacteria in secondary bile acids [9]. Animal studies and cell culture experiments suggest that bile acids can contribute, via nuclear farnesoid X receptor (FXR) and membrane G-protein-receptor (TGR5), to beneficial effects on glucose metabolism [65–67]. Activation of FXR by bile acid or administration of an FXR agonist lowered fasting plasma glucose and improved insulin sensitivity in obese and diabetic mice [68, 69], whereas FXR-deficient mice showed impaired glucose tolerance and decreased insulin sensitivity [68, 70]. TGR5 are expressed at high levels in the small and large intestines and their activation by bile acid stimulates GLP-1 production in an enteroendocrine cell line [71]. Thomas et al. (2009) further showed that administration of a potent TGR5 agonist INT-777 enhances GLP-1 secretion [67].

FOS supplementation modifies the bile acid profile. ScFOS supplementation in mice induces modifications in microbiota composition (*C. coccoides* and *C. leptum* groups), which are correlated to several phenotypic and metabolic parameters, especially to the faecal and blood concentrations of bile acids [46]. Similarly, scFOS intake in humans induces increased concentration of faecal total primary bile acids, associated with higher levels of primary bile acids, but with a decrease in secondary bile acids [72]. Cholesterol may be a blood marker related to biliary acid pattern. However, in our meta-analysis, FOS and OF supplementation did decrease triglyceride concentration in half of the studies whereas results on cholesterol were less consistent. Total cholesterol is not an exhaustive enough parameter to conclude on cholesterol metabolism and informations on LDL- and HDL-cholesterol concentrations would have been necessary to conclude.

The microbiota can also contribute to glucose homeostasis via its impact on inflammatory status. The microbiota is involved in the development of metabolic endotoxemia observed in obese and diabetic individuals or in those consuming a high-fat diet, through the production of inflammatory molecules such as LPS [73]. Microbiota composition modulation after a high-fat diet has been associated with an increased concentration of LPS, in parallel to an alteration of glucose metabolism [74]. In this review, only two studies have analysed the inflammatory profile in parallel to glucose metabolism response. The first one showed a reduction in LPS concentration in ob/ob mice fed a high-fat diet supplemented with prebiotic, associated with changes in microbiota composition [18]. In the second study, scFOS supplementation reduced plasma haptoglobin concentration, a hallmark of inflammation, in obese dogs subjected to a weight loss program [31]. In addition, the team of Cani et al. clearly demonstrated that changes in gut microbiota induced by FOS supplementation decreased inflammatory status in mice with metabolic disorders [75].

To summarize, FOS supplementation improved glucose homeostasis in rodents by mechanisms that could involve their well-known impact on gut microbiota composition towards higher SCFA production, a change in bile acid profile favouring the secretion of GLP-1, and reduced pro-inflammatory compound production. Our bibliography study confirms that dynamic and post-prandial parameters are more efficient for measuring the effects of FOS supplementation, than fasting parameters.

### Implication for human

Our understanding of the mechanisms that control glucose metabolism has benefited from the use of rodent models in metabolic research because similarly to humans, their glucose homeostasis is mainly controlled by insulin release in response to blood glucose and the insulin sensitivity of peripheral organs [76]. Although rodents become glucose intolerant in response to diet-induced obesity over time, they are quite resistant to the development of frank diabetes, that is why researchers relied on pharmacological (e.g. streptozotocin) or genetic models (e.g. Zucker fatty rats) of type 2 diabetes [77]. These models enable to study specific components of glucose intolerance and type 2 diabetes. A wide variety of rodent models were included in our meta-analysis suggesting that improvement of glucose homeostasis by FOS is rather consistent and effective in different conditions. Furthermore, the reduction rates of fasting glycaemia with FOS/OF supplementation observed in rodents would be efficient enough to normalize glycaemia in humans during metabolic disorder development. Indeed, in the case of glucose intolerance, plasma glucose increases by approximately 11% compared to normal glycaemia (> 6.1 mM vs 5.5 mM) and even by 27% in the case of type 2 diabetes mellitus (> 7 mM) [78]. Thus, reducing glycaemia by 10 to 22% with FOS supplementation in the diet as observed in rodents, would be relevant for humans suffering from metabolic disorders to prevent the development of these physiopathological states.

Moreover, rodents may be a good model to study the interplay of gut microbiota changes and development of metabolic diseases in humans, due to their comparable gut physiology and anatomy, and their large extent share of the gut microbiota, not only at the phyla level, but also at the genera level [79–81]. The use of gnotobiotic model is an interesting tool to further increase similarities between animal models and humans and decipher the relationship between gut microbiota and metabolic parameters [46].

It is, however, important to keep in mind that animal models always have some degree of dissimilarity with human physiology. Therefore, results from animal models, including the rodents, are not always directly applicable to humans. Clear differences do exist between species with regard to metabolic regulation [82] and conclusions should be made with caution, especially concerning the conditions under which the effect could be observed in humans, including the daily dose and the minimum duration of dietary supplementation.

Technological advances by using “omics” approaches are enabling scientists to conduct their research in human subjects without using animals in a broad range of disciplines, with non-invasive or minimally invasive techniques. The identification of new biomarkers would allow their common clinical use for diagnosis and monitoring of metabolic disorders.

## Conclusion

In conclusion, this review, by using rodent model, evidenced from different short-term trials, that the use of dietary FOS can be considered as a beneficial dietary intervention for the reduction of circulating postprandial glucose and insulin concentrations in metabolic physiopathology. Some previous studies in rodents correlated these findings with changes in the growth and function of specific gut bacteria.

Long-term prospective trials investigating primary metabolic end points are now required in humans to be able to make practical recommendations.

## Abbreviations

DIO: Diet-induced obesity
DP: Degree of polymerization
FOS: Fructo-oligosaccharides
FSIGTT: Frequently sampled intravenous glucose tolerance test
FXR: Nuclear farnesoid X receptor
GPR: G protein-coupled receptor
LPS: Lipopolysaccharide
OF: Oligofructose
SCFA: Short-chain fatty acid
scFOS: Short-chain fructo-oligosaccharides
TGR-5: Takeda G-protein-coupled receptor 5
